# ETHE1 overexpression promotes SIRT1 and PGC1α mediated aerobic glycolysis, oxidative phosphorylation, mitochondrial biogenesis and colorectal cancer

**DOI:** 10.18632/oncotarget.26958

**Published:** 2019-06-18

**Authors:** Mavee Witherspoon, Davinder Sandu, Changyuan Lu, Kehui Wang, Robert Edwards, Anthony Yeung, Ozkan Gelincik, Giovanni Manfredi, Steven Gross, Levy Kopelovich, Steven Lipkin

**Affiliations:** ^1^Department of Medicine, Weill Cornell College of Medicine, New York, NY, USA; ^2^Department of Pharmacology, Weill Cornell College of Medicine, New York, NY, USA; ^3^Department of Pathology and Laboratory Medicine, University of Irvine School of Medicine, Irvine, CA, USA; ^4^Fox Chase Cancer Center, Philadelphia, PA, USA; ^5^Department of Neurology, Weill Cornell College of Medicine, New York, NY, USA

**Keywords:** familial adenomatous polyposis, ethylmalonic encephalopathy 1, mitochondrial bioenergetics, aerobic glycolysis, colorectal cancer

## Abstract

Ethylmalonic Encephalopathy Protein 1 (*ETHE1*) is a sulfur dioxygenase that regulates cellular H_2_S levels. We previously demonstrated a significant increase of *ETHE1* expression in "single-hit" colon epithelial cells from crypts of patients with Familial Adenomatous Polyposis (FAP). Here, we report elevated levels of *ETHE1* expression and increased mitochondrial density occurring in-situ in phenotypically normal FAP colorectal mucosa. We also found that constitutive expression of *ETHE1* increased aerobic glycolysis ("Warburg effect"), oxidative phosphorylation, and mitochondrial biogenesis in colorectal cancer (CRC) cell lines, thereby depleting H_2_S which relieved the inhibition of phosphodiesterase (PDE), and increased adenosine monophosphate (AMP) levels. This led to activation of the energy sensing AMP-activated protein kinase (AMPKp), Sirtuin1 (SIRT1) and peroxisome proliferator-activated receptor γ coactivator 1α (PGC1α), a master regulator of mitochondrial biogenesis. By contrast, shRNA silencing of *ETHE1* reduced PDE activity, AMPKp/SIRT1/PGC1α levels and mitochondrial biogenesis. Constitutive expression of *ETHE1* accelerated both CRC cell xenograft and orthotopic patient derived xenograft CRC cell growth *in vivo*. Overall, our data nominate elevated *ETHE1 expression levels* as a novel biomarker and potential therapeutic target for the prevention of CRC tumorigenesis.

## INTRODUCTION

The discovery of tumor suppressor genes (TSGs) and their singular role in the etiology of heritable cancers presents the opportunity to study early biomarkers and potential targets during cancer progression [1]. Previously, we investigated gene expression patterns of phenotypically normal “one-hit” cells before they become hemizygous or homozygous for the inherited mutant TSG that promotes tumor formation in hereditary cancers. This approach enables discovery of biomarkers and molecular targets in clinically-identifiable pre-neoplastic lesions such as polyposis of the colon, wherein numerous lesions appear before progression to adenocarcinomas [1–4].

Familial Adenomatous Polyposis (FAP) is characterized by hundreds to thousands of adenomatous polyps throughout the colorectum [5, 6]. The majority of FAP patients carry a germline mutation in the adenomatous polyposis coli (*APC*) tumor suppressor, a negative regulator of WNT signaling and regulator of cell proliferation [7]. The high rate of somatic *APC* mutations in sporadic colorectal cancer is consistent with inactivation of the *APC* protein playing a critical role in the initiation of colorectal cancers [8]. This highlights the need to improve our understanding of events that occur during the transition of normal colon mucosa to cancer including development of early biomarkers and potential targets to prevent/treat FAP and other cohorts at high risk of CRC [9–15].

A recent proteomic study showed that phenotypically normal epithelial cells from the colonic crypts of FAP patients, referred to as “one-hit” cells, when compared to normal colon epithelial cells, revealed a significant increase (~27-fold) of the Ethylmalonic encephalopathy protein 1 (*ETHE1*) which, represented the highest increase in expression among all proteins identified [16]. *ETHE1* is a sulfur dioxygenase, a hydrogen sulfide (H_2_S) catabolic enzyme, that is widely expressed in various tissues and is present in the mitochondria, cytosol and nucleus of eukaryotic animals [17]. In the mitochondria, *ETHE1* facilitates H_2_S catabolism via oxidation and conversion of sulfide quinone reductase generated glutathione persulfides (GSSH) to sulfite (H_2_SO_3_)[18, 19]. Sulfite is further oxidized to sulfate that is secreted extra-cellularly. Consistent with this role, germline bi-allelic *ETHE1* mutations cause ethylmalonic encephalopathy[20-23], a genetic disease in which H_2_S accumulates in critical tissues and can reach concentrations in the brain that inhibit Cytochrome C Oxidase (COX), blocking mitochondrial respiration, increasing lactic acid accumulation, and inducing encephalopathy [22, 24, 25]. Here, we focused on the colon since *ETHE1* is highly expressed in normal colorectal epithelium wherein it modulates the accumulation of toxic endogenous H_2_S generated by colonic microbiota, dietary sulfur containing compounds and endogenous cellular H_2_S produced by cystathionine beta-synthase (CBS) [19, 26].

We found abnormal expression of *ETHE1* and increased mitochondria density in phenotypically normal APC^+/-^ FAP intact colorectal mucosa. Furthermore, using constitutively expressed *ETHE1* in CRC cells we identified novel mechanisms that link augmented *ETHE1* expression with aerobic glycolysis and mitochondria biogenesis. We found that constitutive expression of *ETHE1* reduces H_2_S mediated inactivation of phosphodiesterases (PDEs) and increases AMP concurrent with increased AMPK phosphorylation. This in turn activates the NAD-dependent protein deacetylase Sirtuin 1 (SIRT1). AMPK and Sirt1 are key metabolic sensors that regulate mitochondrial respiration and aerobic glycolysis [27-31]. AMPK and SIRT1 directly activate the nuclear receptor Peroxisome Proliferator-Activated Receptor Gamma Coactivator 1 Alpha (PGC1α) through phosphorylation (AMPK) and deacetylation (SIRT1), respectively [32-34]. PGC1α then drives mitochondrial biogenesis and oxidative phosphorylation leading to increased CRC proliferation, angiogenesis, and tumor growth *in vivo*.

Overall our studies provide new insights into the mechanistic role of elevated *ETHE1* expression levels during CRC tumorigenesis in phenotypically normal “one-hit” FAP colon, highlighting the utility of the "one-hit" model in heritable cancers to facilitate the discovery of potential biomarkers/targets, early during CRC progression.

## RESULTS

### *In situ* expression of *ETHE1* in intact colon mucosa tissue specimens of FAP patients

**Figure 1:**
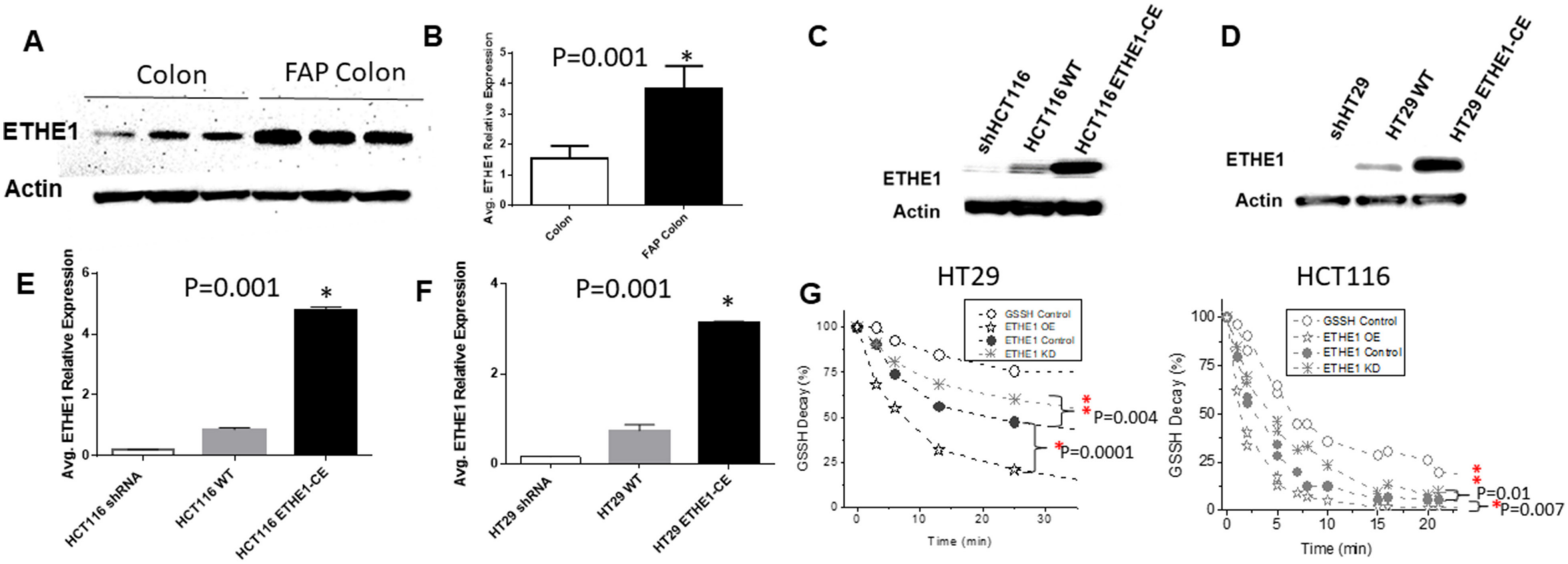
Increased ETHE1 expression and Activity in FAP and CRC. Total protein extracts from FAP and colon biopsy (**A**) (20ug protein per sample) were subjected to immunoblotting with anti-ETHE1 (1:500 Abgent) or anti-Actin( 1:10000 Abcam). (**B**) Avg. relative expression of FAP ETHE1 by densitometry of Western blots(1way ANOVA,N=3,p=0.001). Whole cell lysates from shRNA knockdown, scrambled control(WT) or lentiviral (genecopoeia) generated ETHE1 constitutive expressing (CE) CRC cell lines HCT116(**C**) and (**D**) HT29. Avg. relative expression of ETHE1 CRC variants (**E**) HCT116 and (**F**) HT29 by densitometry of Western blots ; (p=0.001 unpaired t-test). (**G**) Kinetic analysis of ETHE1 catabolism of GSSH in HT29 and HCT116 ETHE1-CE lysate. To determine ETHE1 activity, whole cell lysates were incubated with glutathione persulfide (GSSH), ETHE1 increases the rate of O_2_ dependent consumption of glutathione persulfides (GSSH). ^*^P-values <0.01.

Yeung et al. demonstrated that phenotypically normal, non-transformed APC "single-hit" cells in FAP epithelium showed the highest differential increase in *ETHE1* levels among all proteins identified compared to normal colon epithelial cells from control subjects [16]. We further validated these findings using phenotypically normal intact colon mucosa surgical specimens from FAP (N=3) patients carrying APC c.388delA, APC c.1240delC and APC c.2586insCA truncating mutations, and individuals with no history of CRC (N=3) who were matched for location (sigmoid colon) age, gender and ethnicity, collected during screening colonoscopy. Western blot analysis confirmed >160% (p=0.001 unpaired t-test) increase in *ETHE1* protein levels in FAP colonic tissues (Figure 1A and 1B).

### *ETHE1* expression and activity in CRC cell lines

To study the role of increased *ETHE1* levels in CRC, we used human-derived HCT116 and HT29 CRC cell lines that stably constitutively expresses a full-length cDNA encoding *ETHE1*. Concurrently, gene specific short-hairpin RNA (shRNA) sequences were used to knock-down *ETHE1* in these cell lines. Western blot (Figure 1C and 1D) and densitometry analysis (Figure 1E and 1F) confirmed increased *ETHE1* protein levels (p=0.001 unpaired t-test) in constitutively expressed HCT116 and HT29 (Figure 1E and 1F) cells.

Next, we tested whether *ETHE1* was enzymatically active in CRC cells. *ETHE1* facilitates H_2_S catabolism via oxidation of sulfide quinone reductase generated glutathione persulfides (GSSH) to sulfite (H_2_SO_3_), in a reaction that requires oxygen and water. Sulfite is further oxidized to sulfate and secreted. Several studies have shown that GSSH is the main substrate for *ETHE1*[35-38]. Therefore, we mixed freshly prepared 735 μM glutathione persulfide (GSSH) with 200 μg cell lysate under air-saturated conditions at pH 7.4 and measured the disappearance rate of GSSH using an established monobromobimane (MBB) method with LC/MS [35]. In both ETHE1 overexpressing CRC cell lines, metabolism of GSSH was increased compared to *ETHE1* scrambled control cell lysates (HT29-CE p=0.0001; HCT116-CE p=0.007) (Figure 1G). Conversely, *ETHE1* knockdown CRC cells showed reduced rates of GSSH consumption relative to controls. Thus, *ETHE1* actively reduces H_2_S metabolites in CRC cell lines.

### *ETHE1* protein levels correlate with FAP mitochondrial protein enrichment

We performed a re-analysis of 214 differentially expressed two-dimensional gel protein spots identified in morphologically normal, “single-hit” FAP and low CRC risk colon epithelial cells datasets [16]. Using this approach, we identified 66 non-redundant proteins with a threshold of > 1.5X up-regulation (p<0.01) in the "single-hit" FAP cells. The annotation cluster with the highest enrichment (enrichment score 14.95) for *ETHE1* upregulation in these cells was identified as “Mitochondrial proteins” (Supplementary Table 1). This subset of upregulated proteomic annotation cluster categories included mitochondrial transit peptide containing proteins (p=1.62E-15), mitochondrial lumen and matrix proteins (p=7.63E-15), as well as envelope (outer membrane) and mitochondrial inner membrane proteins (Supplementary Table 1).

**Figure 2:**
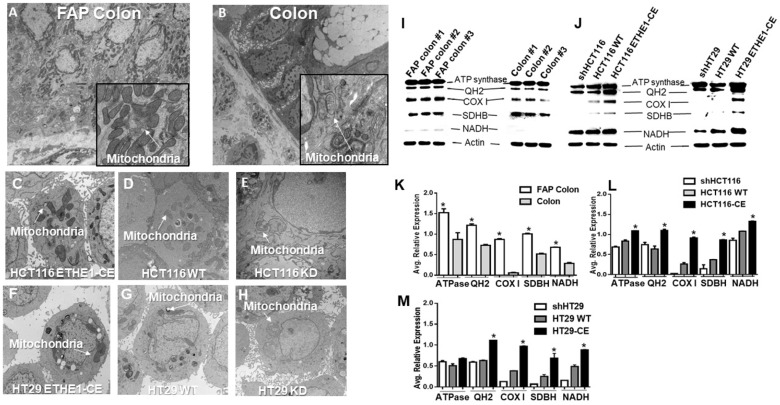
ETHE1 promotes expression of CRC mitochondrial oxidative phosphorylation proteins. Representative electron micrographs of biopsied patient mucosa, shows increased mitochondrial matrix density in (**A**) intact FAP colon vs (**B**) intact colon (n=3). Constitutive expression of ETHE1 in CRC cells HCT116(**C**), HT29 (**F**) displays increased mitochondrial matrix density verses scrambled controls (**D, G**). A decrease in mitochondrial matrix density was observed in ETHE1 shRNA knockdownHCT116 (**E**) and (**H**) HT29 CRC cells. Original magnification; 8000x to 50,000x, Arrows indicate mitochondria. Analysis of 20 images per group. ^*^Two-sided Paired t-test p<0.001. Total protein extracts from FAP colon and colon biopsy (20ug/samples) (**I**), ETHE1 overexpressing HCT116 and HT29 (**J**) probed for mitochondrial OXPHOS proteins (MitoProfile Total OXPHOS; abcam). Avg relative OXPHOS protein expression of FAP colon vs (**K**) colon, ETHE1 CRC variants HCT116 (**L**), HT29 (**M**) determined by densitometry of Western blots; (1way ANOVA,N=3,p=0.002).

To test further mitochondrial involvement, we compared the ultrastructural characteristic of phenotypically normal, intact FAP colon tissue specimens, with matched colon tissues from control individuals by transmission electron microscopy (EM). Phenotypically normal FAP colon epithelial biopsies showed densely distributed mitochondria throughout the cytoplasm and increased mitochondrial matrix staining, when compared to intact colon controls (Figure 2A and 2B). Next, we examined CRC cell lines with different *ETHE1* expression levels for their ultrastructural features. Similar to intact FAP colon biopsies, ultrastructural analysis of constitutively expressing *ETHE1* HCT116 (Figure 2C and 2D) and HT29 cells (Figure 2F and 2G) showed increased mitochondrial matrix staining. In contrast, silencing of *ETHE1* reduced mitochondrial matrix density in HCT116 (Figure 2E) and HT29 (Figure 2H) cells, compared with scrambled controls. Overall, we show a strong correlation between elevated *ETHE1* levels and increased mitochondrial density and matrix in histologically normal colonic epithelia from FAP patients and in CRC cells.

### *ETHE1* upregulates oxidative phosphorylation in CRC

Since the mitochondrial matrix plays a critical role to convert energy derived from oxidation/reduction reactions of electron transport, we examined the expression levels of key mitochondrial oxidative phosphorylation (OXPHOS) related proteins in whole cell homogenates that were derived from phenotypically normal biopsied tissue specimens of FAP patients and control individuals. Phenotypically normal FAP patient lysates exhibited increased (P=0.001; 1-way ANOVA) levels of ATP synthase (*ATPase*) subunits, succinate dehydrogenase (*SDBH*), cytochrome c reductase (*QH2*), cytochrome c oxidase (*COX*) and ubiquinone oxidoreductase (NADH) (Figure 2I) protein levels as compared with low CRC risk controls. Previous studies have shown higher *COXI* expression in CRC cells than in normal colon epithelia, indicating a role for increased cytochrome c oxidase (*COX*) expression in the transformation of colon epithelia [39]. Consistent with these reports, densitometric analysis of FAP vs sporadic western blot data, showed a ~15 fold increase in *COX* expression (paired t-test,p=0.001) in the phenotypically normal intact FAP colon biopsies (Figure 2K).

To investigate whether *ETHE1* levels were directly associated with this subset of mitochondrial protein expression, we probed constitutively expressing *ETHE1* CRC cell lines for OXPHOS levels. Analysis of *ETHE1* CRC cell homogenates showed that constitutive upregulation of *ETHE1* in HCT116 and HT29 cells increased OXPHOS protein levels (Figure 2J). Densitometric analysis revealed that upregulation of *ETHE1* in HCT116 (Figure 2L) and HT29 (Figure 2M) significantly increased (>2x, p=0.001) expression of *COXI*, *SDBH* and *NADH*. This effect was attenuated by shRNA-mediated silencing of ETHE1 CRC cells, resulting in approximately a 3x to 10x fold reduction (P<0.05) of COXI expression in HT29 and HCT116 (Figure 2L and 2M), respectively. Taken together, these results are consistent with a direct role of ETHE1 to drive mitochondrial OXPHOS protein expression and mitochondrial content.

### Overexpression of *ETHE1* enhances CRC mitochondrial respiration and aerobic glycolysis

**Figure 3:**
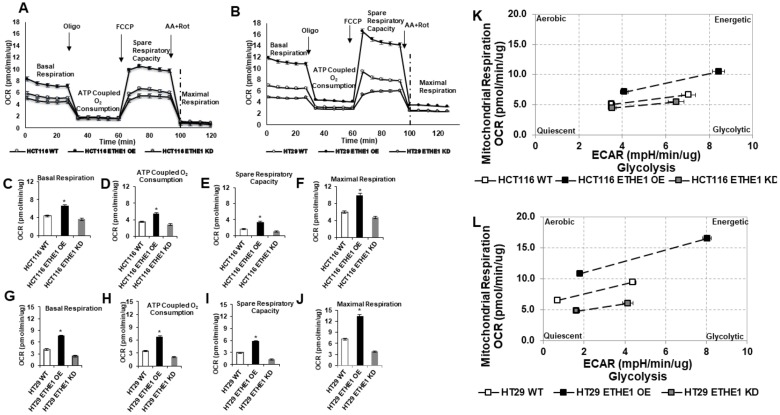
Overexpression of ETHE1 enhances CRC mitochondrial respiration. Oxygen Consumption rate (OCR) in shRNA knockdown or lentiviral ETHE1 constitutive expressing (CE) CRC cell lines (**A**) HCT116 and (**B**) HT29. Constitutive expression of ETHE1 significantly increased, whereas silencing of ETHE1 reduced HCT116 and HT29 basal OCR (**C, G**), ATP Coupled O_2_ Consumption (**D, H**), spare respiratory capacity (**E, I**) and maximal respiration (**F, J**). ^*^P < 0.05 or ^**^P < 0.01 vs. wt control. Data represent mean ± SEM of n = 4. two-sided Student's *t* test. OCR determined by; Basal respiration (rate before first injection – non-mitochondrial respiration rate), ATP Coupled O_2_ Consumption (rate before oligomycin injection – rate after oligomycin injection), Maximal Respiration (rate after FCCP– non-mitochondrial respiration rate), Spare Respiratory Capacity (Maximal Respiration – Basal respiration). ETHE1 constitutive expression in (**K**) HCT116 and HT29 (**L**) CRC cells show an increased glycolytic response under stress (oligomycin).

Our results showed that phenotypically normal, FAP colon biopsies and CRC cell lines contained large numbers of mitochondria with increased mitochondrial matrix staining, consistent with metabolically highly active mitochondria. To investigate the role of elevated *ETHE1* levels as a driver of mitochondrial respiration, we used the Seahorse XF96 extracellular flux analyzer on CRC cells with elevated, normal and knocked-down levels of *ETHE1*. Using this approach, we generated mitochondrial bioenergetics profiles from HCT116 (Figure 3A) and HT29 (Figure 3B) cells. Constitutive expression of *ETHE1* significantly increased HCT116 basal respiration (~51%), ATP turnover (~57.5%), spare respiratory capacity (~113%) and maximal respiration (~67.7%) (Figure 3C-3F) compared with scrambled parental control cells. It also increased HT29 basal respiration (~84%), ATP turnover (~93.9%), maximal respiration (~88.1%) and spare respiratory capacity (~95.5%) (Figure 3G-3J). Notably, all CRC cell lines, regardless of *ETHE1* expression levels demonstrated a positive association between increased oxygen consumption rate (OCR) and extra-cellular acidity rate (ECR), indicating the concurrent occurrence of enhanced mitochondrial respiration and aerobic glycolysis ("Warburg effect") (Figures 3K and 3L). Yet this association appeared to trend most clearly in the case of constitutively enriched *ETHE1* cell lines (Figures 3K and 3L). In this context, shRNA silencing of *ETHE1* reduced basal respiration, ATP turnover, spare respiration capacity, and maximal respiration. Altogether, our data demonstrate that upregulation of *ETHE1* increased not only protein levels of key mitochondrial oxidative phosphorylation related proteins, but has also contributed to metabolic adaptations associated with tumor malignancies wherein both aerobic glycolysis and respiratory capacity are implicated[40].

### *ETHE1* regulates the SIRT1/PGC1α axis

The mechanisms responsible for the regulation of mitochondrial biogenesis and function involve complex networks of transcriptional factors and cofactors. The Sirtuin (*Sirt*) family of deacetylases activates mitochondriogenic cellular programs and increased aerobic glycolysis [28, 30, 32, 41]. Sirtuin1 (*SIRT1*) is a well characterized NAD dependent type III nuclear deacetylase. *SIRT1* transcriptionally regulates the peroxisome proliferator-activated receptor γ coactivator 1α (*PGC1α*) [29, 30, 32, 33], an important regulator of mitochondrial biogenesis and function [33, 42].

**Figure 4:**
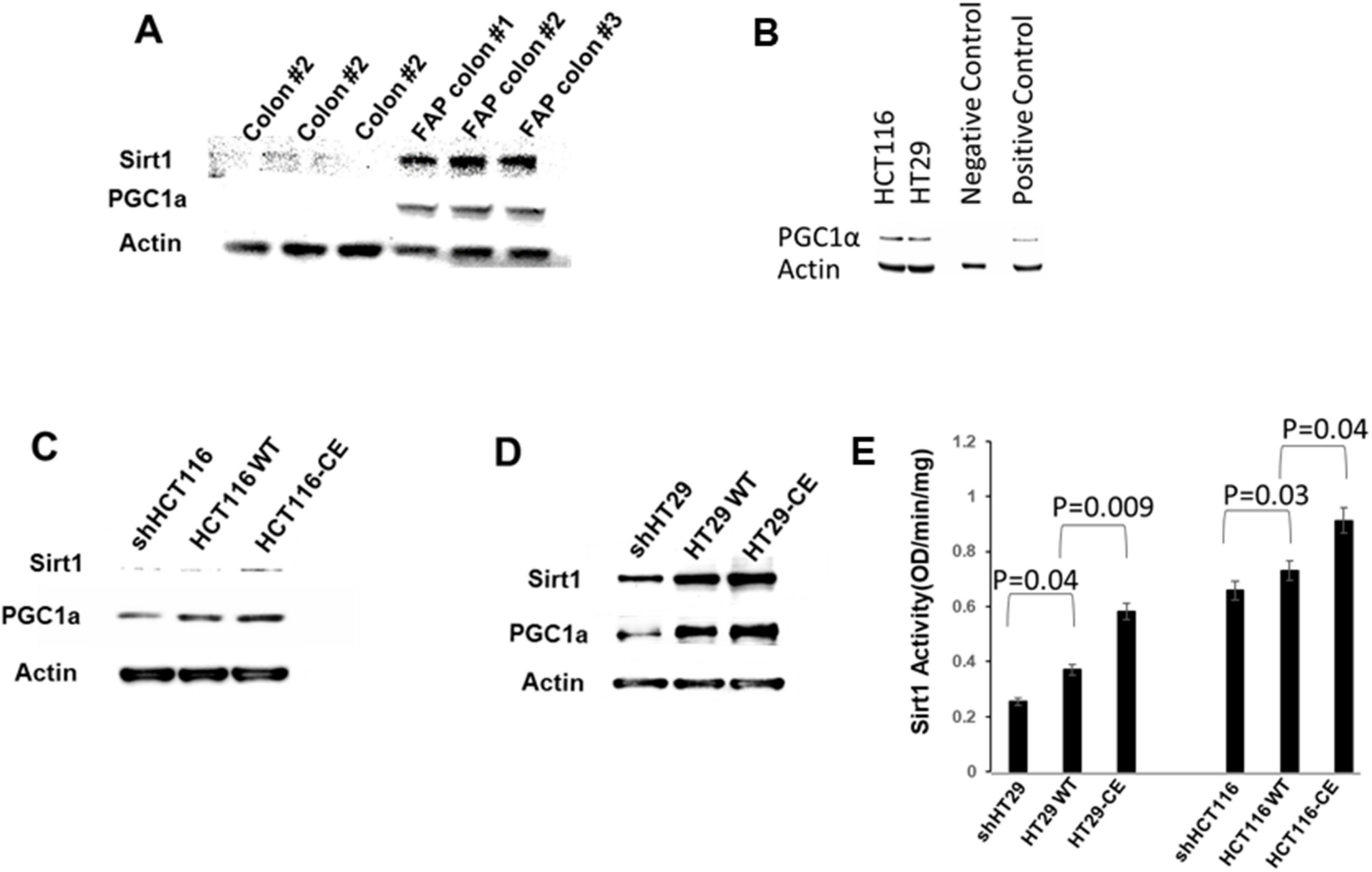
ETHE1 regulates the Sirt1/PGC1α axis. (**A**) Total protein extracts from FAP colon and colon biopsy (20ug/samples) (**B**) Total protein extracts from HCT116 or HT29 shRNA controls, probed for PGC1a (abcam 1:1000). Positive and negative control lysates included (Novus biologicals). (**C**) ETHE1 overexpressing HCT116 and (**D**) HT29 CRC cells showed increased SIRT1 and PGC1α protein levels compared with scrambled and shRNA controls (Abcam 1:500 Sirt1, Abcam 1:1000 PGC1α). (**E**) ETHE1 overexpressing HT29 and HCT116 CRC cells (5ug of nuclear extract/sample) shows increased Sirt1 activity (Abcam). N=3, Statistics; Paired t-test. Two-sided.

First, we therefore determined *SIRT1* and *PGC1α* protein levels in cell homogenates from phenotypically normal, intact FAP colon tissue biopsies. *SIRT1* and its target *PGC1α* protein levels were significantly increased in FAP colon verses normal colon controls (Figure 4A). Next, we determined PGC1 expression in HCT116 and HT29 scrambled shRNA control cells (Figure 4B). We then examined constitutively expressing *ETHE1* HCT116 (Figure 4C) and HT29 (Figure 4D) cells to test whether SIRT1 and PGC1α levels were directly regulated by *ETHE1*. Similar to intact FAP tissue biopsies, SIRT1 and PGC1α were upregulated in constitutively expressed *ETHE1* cell lines. In contrast, shRNA silencing of *ETHE1* reduced both PGC1α and SIRT1 protein levels in CRC cells.

In addition, we determined *SIRT1* deacetylase activity in high and low ETHE1 expressing CRC cells. We prepared nuclear extracts from constitutively expressing *ETHE1* HCT116 and HT29 cells using a SIRT1 activity assay kit (Figure 4E). The results showed that, consistent with increased *SIRT1* levels, SIRT1 functional deacetylase activity was also upregulated in high *ETHE1* expressing HCT116 and HT29 cells. In contrast, shRNA silencing of *ETHE1* reduced SIRT1 activity (Figure 4E). Thus, ETHE1 levels can positively drive SIRT activity.

### PDE mediated stimulation of pAMPK/SIRT1 is linked to *ETHE1* levels

**Figure 5:**
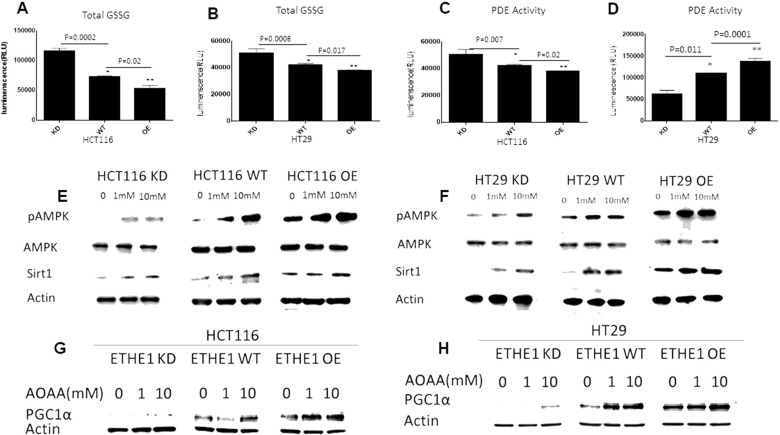
ETHE1 levels drive PDE mediated stimulation of pAMPK/Sirt1. Cells were seeded (30K) in 96 well plates, HCT116 (**A**) and HT29 (**B**) cells expressing low (KD), scrambled control (WT) or high ETHE1 (OE) were used to measure H_2_S, as determined by GSSG levels (GSH/GSSG Promega), ETHE1 high expressing cells showed reduced GSSG, suggesting reduced H_2_S levels. shRNA silencing of ETHE1 showed increased GSSH levels; N=3; unpaired T-test. PDE activity determined by cAMP-Glo Assay (Promega), (cAMP levels are inversely proportional to luminescence); HCT116 (**C**) and HT29 (**D**) ETHE1 overexpressing cells (seeded 30K in 96 well plates) show reduced cAMP levels (high luminescence), suggesting increased PDE activity, shRNA silencing of ETHE1 increased cAMP levels, as compared with wild type control. N=3; unpaired T-test. Total protein extracts from HCT116 (**E**) and HT29 (**F**) CRC cells treated with the CBS inhibitor aminooxyacetic acid (AOAA), showed increased activation of AMPK (pAMPK) and Sirt1 expression. PGC1α levels in HCT116(**G**) and HT29(**H**) ETHE1^KD/WT/OE^ CRC cells treated with AOAA.

H_2_S inhibits phosphodiesterase activity, affecting the rate of c-AMP conversion to AMP [34, 43, 44]. Building upon our findings that overexpression of *ETHE1* increased the rate of GSSH catabolism in CRC cell lysates (Figure 1G), we then determined whether H_2_S levels can alter phosphodiesterase activity. We first measured the levels of total GSSH in constitutively expressing *ETHE1* HCT116 and HT29 using the GSSG-Glo assay. Consistent with increased *ETHE1* -mediated H_2_S catabolism, H_2_S levels were decreased in high *ETHE1* expressing HCT116 (Figure 5A) and HT29 (Figure 5B) cells, as determined by GSSG levels. In contrast, shRNA-mediated silencing of *ETHE1* CRC upregulated GSSH levels. Next, we determined PDE activity in high and low *ETHE1* expressing cells using a cAMP-Glo assay. These results showed that cAMP levels were decreased in high *ETHE1* expressing HCT116 (Figure 5C) and HT29 cells (Figure 5D), consistent with increased PDE activity and conversion of cAMP to AMP.

To study the mechanism through which *ETHE1* decrease of H_2_S promotes the AMPK/SIRT1 axis, including increased mitochondrial biogenesis and function, we used a Cystathionine-β-synthase (CBS) inhibitor, aminooxyacetic acid (AOAA). *CBS* is a H_2_S producing enzyme, shown to be overexpressed in CRC cells [26, 45]. We, therefore, prepared total cell extracts from constitutively expressing *ETHE1* HCT116 and HT29 cells treated with AOAA (1-10mM) and measured AMPKp and SIRT1 protein levels. We found that inhibition of *CBS* activity resulted in increased AMPKp, SIRT1 and PGC1α expression in these cells (Figure 5E-5H). Overall, these results are consistent with increased *ETHE1* catabolism of endogenous H_2_S, leading to increased PDE activity and AMPKp/SIRT1 expression. Since Cystathionine-β-synthase (*CBS*) is an important contributor to intracellular H_2_S production (24), we also examined whether low or high *ETHE1* levels altered *CBS* expression in CRC cells. Western blot analysis showed that changes in *ETHE1* levels had no effect on *CBS* expression in HT29 (Supplementary Figure 1A) or HCT116 cells (Supplementary Figure 1B). Therefore, increased *ETHE1* levels do not appear to notably affect the CBS pathway.

### *ETHE1* stimulates oncogenesis of CRC cells *in vitro* and *in vivo*

**Figure 6:**
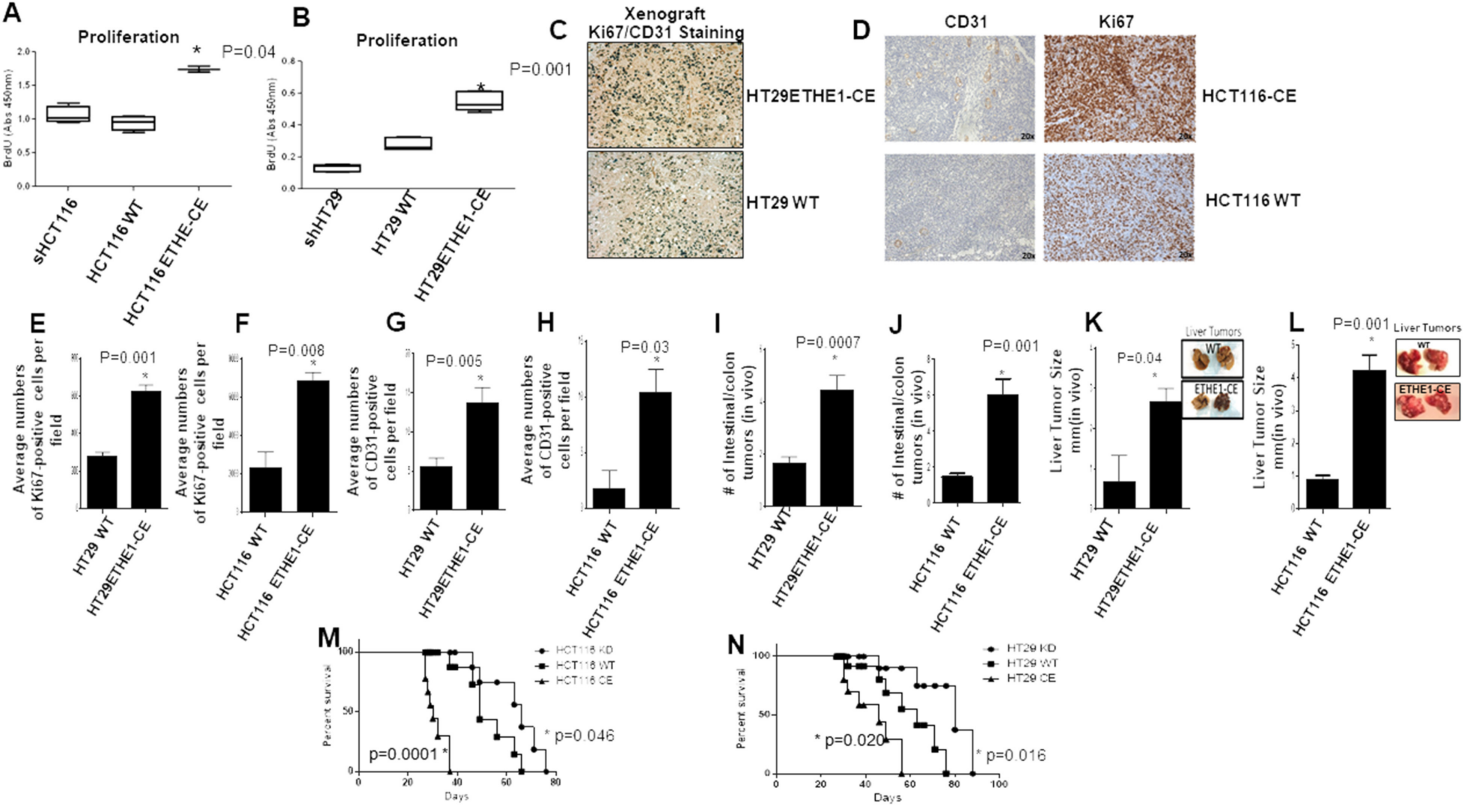
ETHE1 stimulates CRC tumorgenicity *in vivo*. (**A**) HCT116 and (**B**) HT29 CRC cell proliferation, 50K cells plated. Cells were incubated with BrdU (BrdU cell proliferation assay, Cell Signaling). N=3. Abs 450. two-sided Student's *t* test. **(C** and **D)** HT29/HCT116 CD31/Ki67 staining of xenografts (CD31, Ki67 Cell signaling), shRNA controls were used. Quantification of Ki67/CD31 positive cells HT29^shRNAwt/CE^ and HCT116^shRNAwt/CE^ (**E-H**) showed increased staining in ETHE1 high expressing xenografts, 20x magnification images (N=3) analyzed using ImageJ software, Statistics; Paired t-test. Two-sided. Tail vein injection of CRC cells into NOD/SCID mice showed increased intestinal/colon tumor numbers **(I** and **J)** and extracolonic **(K** and **L)** tumor size was determined (N=8). Reduce survival in NOD/SCID mice injected with ETHE1 expressing (**M**) HCT116^ETHE1-CEE^ (**N**) HT29^ETHE1-CE^ cells. Increased survival in mice injected with HCT116^ETHE1-KD^ or HT29^ETHE1-KD^ (log rank test) (N=8;mice per group).

Increased expression of SIRT1 and PGC1α increase cell proliferation and angiogenesis [46, 47]. Since *ETHE1* upregulated SIRT1 and PGC1α, we tested whether cell proliferation was affected by ETHE1 expression levels using BrdU incorporation assays. The results show that high expressing *ETHE1* HCT116 (Figure 6A) and HT29 (Figure 6B) cells exhibit increased cell proliferation *in vitro*.

Next, we performed xenograft studies to test the effects of increased *ETHE1* expression on angiogenesis. Male NOD/Scid mice were injected in either flank (S.C) with constitutively expressing *ETHE1* HT29/HCT116 or scrambled control cells. The percentage of KI67 positive tumor cell nuclei was counted in 4 × random images (magnification 20x) (Figure 6C, 6D). The results show that, in contrast to scrambled controls, high *ETHE1* expression significantly increased HT29 and HCT116 Ki67^+^(122-194%) staining (Figure 6E and 6F). In addition, we found that high *ETHE1* expression significantly increased the number of vascular endothelial CD31+ cells (Figure 6G and 6H) in xenograft tumors by >2 fold. Taken together, these data are consistent with a role for *ETHE1* to increase not only CRC cell proliferation, aerobic glycolysis and OxPhos, but also angiogenesis *in vivo*.

To assay the effects of high *ETHE1* expression on CRC tumorigenicity, we injected (via lateral tail vein) male non-obese diabetic/severe combined immunodeficient (NOD/SCID) mice with constitutively expressing *ETHE1* HCT116 (1x10^6^) or HT29 (1x10^6^) cells. High *ETHE1* expression increased intestinal and extra-colonic HCT116 and HT29 tumor multiplicity and tumor size (4x fold) compared with scrambled control parental cells (Figure 6I-6L). Importantly, mice injected (via lateral tail vein) with high expressing *ETHE1* HCT116 or HT29 cells showed reduced overall survival (Figure 6M and 6N). These results are consistent with *ETHE1* levels as a mechanistic driver of colorectal cancer growth *in vivo*.

## DISCUSSION

Previous studies have shown that germline *ETHE1* mutations in ethylmalonic encephalopathy lead to an accumulation of H_2_S and inhibition of Cytochrome C Oxidase (COX) which is essential for mitochondrial respiration, affecting severe dysfunction in cellular energy metabolism [18, 48, 49]. While the effects of *ETHE1* deficiency have been thoroughly investigated, the consequences of increased *ETHE1* expression in CRC tumorigenesis are poorly understood. In hepatocellular carcinoma (HCC), *ETHE1* is overexpressed [50], where it is associated with inhibition of caspase 9 activation, suppressing DNA-damage induced apoptosis by binding to the RelA p65 subunit of NFkB, and increasing its export from the nucleus [50]. However, in our initial studies we did not see activation of this pathway in phenotypically normal intact colon mucosa from FAP patients (cancer initiation), indicating that increased *ETHE1* expression involves different mechanisms in different cancer types.

Similar to previous proteomic studies on “single-hit” FAP/APC normal colon epithelial cells [16], our data show a significant increase in f *ETHE1* expression in phenotypically normal intact mucosa tissue from FAP patients, including an associated increase in mitochondrial content/matrix, as compared with normal colon mucosa from control subjects. The impact of *ETHE1* overexpression in early *in situ* CRC tumorigenesis on mitochondrial proteins was further illustrated through re-analysis of differentially expressed two-dimensional gel protein spots identified in phenotypically normal, “single-hit” FAP epithelial cells[16]. Using this approach, we identified 66 non-redundant proteins in “single-hit” FAP epithelial cells in which the annotation cluster with the highest enrichment (enrichment score 14.95) in these cells was identified as “Mitochondrial proteins” (Supplementary Table 1), attesting to the strong correlation between elevated *ETHE1* levels and increased mitochondrial proteins in “single-hit” FAP cells. These data are consistent with our current observations regarding *ETHE1* over expression and increased mitochondrial biogenesis seen in phenotypically normal FAP mucosa specimens occurring *in situ*.

Accordingly, we investigated the metabolic impact of *ETHE1* expression in CRC cell lines at baseline and after knockdown or constitutive overexpression. Increased *ETHE1* expression in CRC cells stimulated mitochondrial biogenesis/bioenergetics and increased mitochondrial matrix staining, consistent with an overall increased mitochondrial activity. Thus, constitutively upregulated *ETHE1* in CRC cells share phenotypic similarities with alterations seen in FAP patient normal mucosa epithelium *in situ*. By contrast, mitochondrial matrix, OXPHOS protein expression and respiration levels were reduced following shRNA silencing of ETHE1 in CRC cells. These data are consistent with a novel mechanistic role for ETHE1 function in which, in addition to H_2_S catabolism, if abnormally expressed, it promotes mitochondrial biogenesis and cellular respiration.

**Figure 7:**
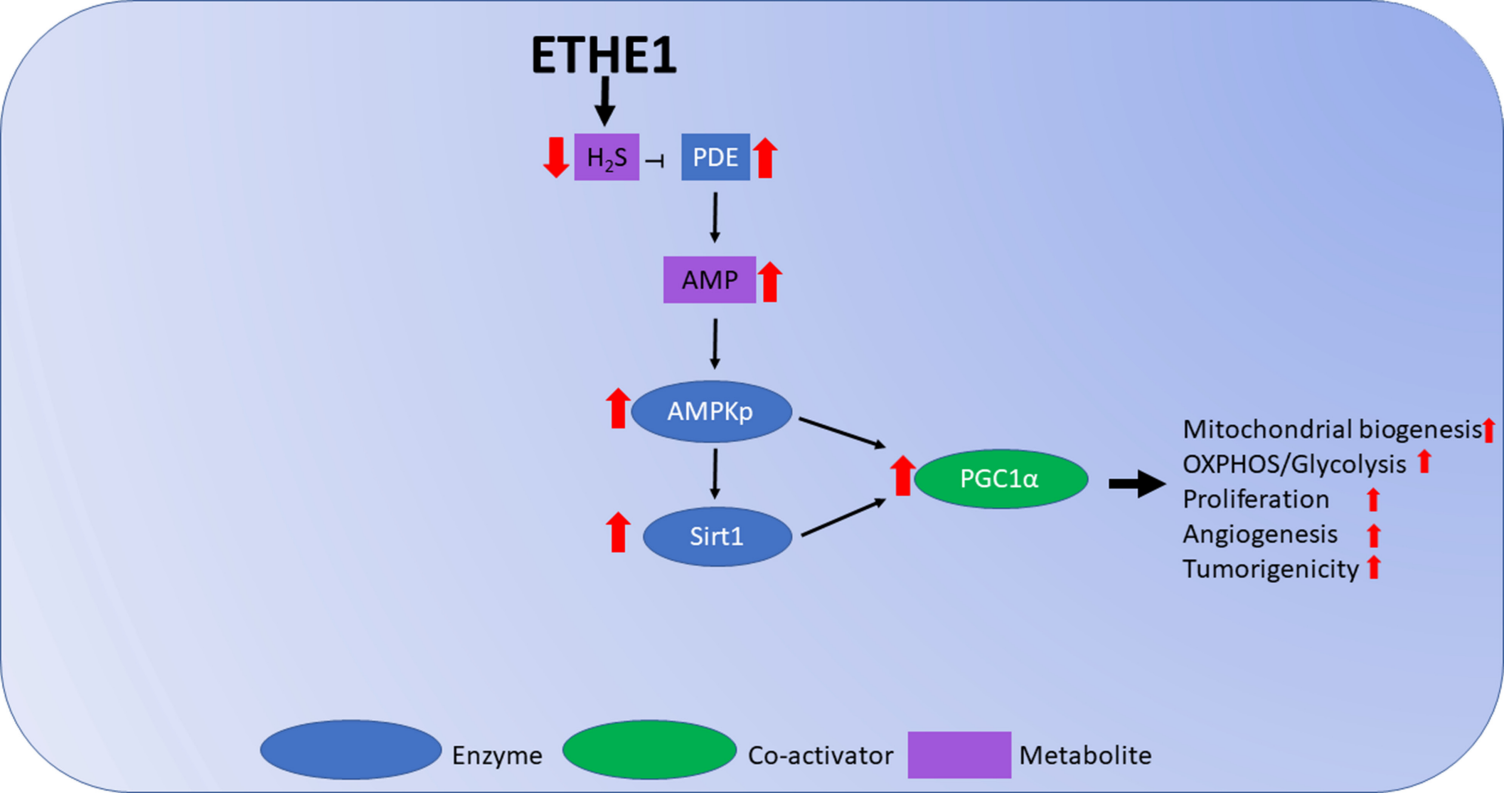
Model figure of ETHE1 roles in FAP. Overexpression of ETHE1 prevents H_2_S mediated inhibition of PDE, increasing AMPKp activation of Sirt1 and subsequent induction of PGC1α, increasing CRC mitochondrial OXPHOS, proliferation, angiogenesis and tumorigenicity.

Mitochondria are key metabolic and biosynthetic regulators that are intimately involved in cancer cell growth. SIRT1, AMPK and PGC1α are important regulators of mitochondrial biogenesis. The NAD+ dependent deacetylase SIRT1 and AMPK have been shown to upregulate PGC1α activity [27, 29, 31, 32, 43]. Thus, our data point to a basic pathway that underlies *ETHE1* overexpression in CRC cells. Our data support a process whereby increased H_2_S catabolism lowers H_2_S levels, which increases PDE activity. This leads to increased AMP, which promotes phosphorylation of AMPK, leading AMPKp and SIRT1 directly to stimulate PGC1α. Together, AMPKp and SIRT1 increase PGC1α and promote CRC mitochondrial biogenesis and respiration, including aerobic glycolysis (the “Warburg hypothesis”) (Figure 7). In fact cellular respiration and aerobic glycolysis have been shown to occur concurrently in many tumors, and although aerobic glycolysis is an inefficient way to generate ATP, most cancer cells depend on it [40]. Supporting this notion are recent studies showing that proliferating cells require metabolic pathways that incorporate nutrients into biomass, and that cancer-associated mutations enable cancer cells to support proliferation rather than efficient ATP production [40, 51-53]. We showed a positive association between increased aerobic glycolysis and cell respiration that is further impacted by *ETHE1*. Thus, *ETHE1* alters the metabolism of cancer cells in order to facilitate increased biomass and cell growth, increased cellular respiration notwithstanding.

Concordant with our results *in vitro*, *in vivo* studies showed that constitutive expression of ETHE1 in CRC cells increased KI67^+^/CD31^+^ staining in NOD/SCID xenograft mouse models. Additionally, mice injected (via lateral tail vein) with constitutively expressing *ETHE1* HCT116 (1x10^6^) or HT29 (1x10^6^) cells show reduced median survival time, increased tumor incidence, tumor burden, multiplicity and metastasis *in vivo*. Significantly, ETHE1 null (KO) CRC cells showed much reduced tumor cell growth and increased median survival time *in vivo* compared to WT or *ETHE1* overexpressing CRC cells. These data provide evidence that upregulation of *ETHE1* plays an important role in driving pathogenesis of CRC *in vivo*.

Previously, ETHE1 protein levels were shown to be elevated in FAP normal colonic mucosa (15). Our findings here confirm these original findings in additional FAP colon biospecimens and extend our current mechanistic understanding of the role of ETHE1 in FAP CRC tumorigenesis with both *in vitro* and *in vivo* studies, leading to the following salient conclusions: First, because *ETHE1* promotes CRC cell growth, and is upregulated in phenotypically normal APC^+/-^ FAP colon epithelium, high *ETHE1* levels may be useful as an early biomarker of CRC premalignant risk. Second, *ETHE1* may be a potential chemoprevention target for FAP and high-risk sporadic CRC patients. Third, there has been a significant amount of interest regarding the potential influence of gastrointestinal microbiota on CRC tumorigenesis (50-54). Specifically, microbial species associated with CRC risk include *fusobacteria*, a genus of anaerobic gram-negative bacteria, and in particular fusobacterium nucleatum which produces H_2_S [54-58]. Thus, paracrine H_2_S production by colonic epithelium adherent *Fusobacteria nucleatum* driving ETHE1 upregulation may constitute an additional mechanism of CRC tumorigenesis for this important bacterium.

## MATERIALS AND METHODS

### Cell culture

Cells (HCT116, HT29) were maintained in Dulbecco’s Modified Eagle Medium (DMEM; Invitrogen Corporation, Carlsbad, CA), 10% fetal bovine serum (Hyclone Laboratories, Inc., Logan, UT), 5 μg/ml gentamicin (Life Technologies, Gaithersburg, MD), and 0.75 μg/ml fungizone antimycotic (Invitrogen Corporation; DMEM complete). All cell lines were incubated in a 95% air and 5% CO_2_ humidified atmosphere at 37°C, and the medium was replaced every 48 h.

### ETHE1 overexpressing CRC lines

Constitutive expression of ETHE1 in HT29 and HCT116 cells, shRNA knockdown and scrambled shRNA control cells were established using a Lentivector-based system according to the manufacturer's instructions (GeneCopoeia Inc., Rockville, MD, USA). For lentiviral-mediated gene transfer, Lv-101 T298) expression vector and transfected into HEK293 cells for pseudoviral packaging. Supernatants were collected and purified viral particles were titered and used to infect cells.

### FAP tissue specimens

Phenotypically normal sigmoid colonic epithelium from FAP patients matched patient age, gender and ethnicity (European ancestry) with patients having screening colonoscopy with no personal, first or second degree history of CRC were obtained from the New York Presbyterian Hospital Center for Advanced Digestive Care Biobank under an IRB approved protocol (0908010582).

### Western blotting and AOAA treatment

For immunostaining, cells lysed in (500 mM NaCl, 50 mM Tris-HCl pH 7.5, 0.5% Triton X-100, 1 mM EDTA, and 1 *μ*M DTT), clarified by centrifugation. 30ug of lysates were loaded on 10%BisTris gels and transferred to PVDF. Membranes were incubated with primary antibody (Anti-ETHE1 sigma, Anti-OxPhos Santa Cruz, Anti-CBS Santa Cruz, Anti-PGC1α Santa Cruz) overnight at 4 °C and secondary antibody for 1 h at 4 °C. For AOAA studies, cells were exposed to 1mM-10mM AOAA for 1hr. Lysates were collected in NP-40 lysis buffer (Boston Bioproducts) supplement with complete mini EDTA free protease inhibitor (Roche). Membranes were incubated with primary antibodies; AMPK, AMPKp, SIRT1 antibodies (Cell Signaling).

### Metabolic flux assay

Cells seeded in triplicate in 100 μl DMEM at a density of 1 × 10^4^/well in XF96 96-well cell culture plate (Seahorse Bioscience, North Billerica, MA) and incubated at 37°C for 24hrs. The XF96 sensor cartridge (Seahorse Bioscience) was prepared by incubation of each sensor pair in 1 ml of Seahorse Bioscience XF96 Calibrant pH 7.4 (Seahorse Bioscience) at 37°C without CO_2_ for 24 h. Prior to analysis, the medium was removed and the wells washed with 1 ml of pre-warmed Seahorse DMEM supplemented with 5 mM glucose. A final volume of 180 μl assay medium was added to each well and the plate incubated at 37°C without CO_2_for 1 hr. Either 1.5 μM carbonyl cyanide-p-trifluoromethoxyphenylhydrazone (FCCP), 5 μM oligomycin (Sigma-Aldrich, St. Louis, MO) or 5 μM antimycin was added to injection ports of the XF96 sensor cartridge in assay medium, equilibrated at least 15 min prior to analysis at 37°C without CO_2._ The XF96 sensor cartridge was calibrated in the Seahorse XF96 analyzer (Seahorse Bioscience), which was pre-warmed to 37°C. Following calibration, the cell culture plate was placed in the analyzer and the extracellular acidification rate (ECAR) and the oxygen consumption rates (OCR) were simultaneously measured via the following protocol: three cycles of mix (2 min), delay (2 min); seven cycles of measure (4 min), mix (2 min), delay (2 min); port A injection; six cycles of mix (2 min), delay (2 min), measure (4 min). Post-drug injection values were normalized to respective basal value and expressed as either mpH/min/10^4^ cells (ECAR) or pmoles/min/10^4^ cells (OCR).

### Animals

All animal studies and procedures done in accordance with protocols approved by WCMC. NOD/SCID mice purchased from Jackson Laboratory. CCIC 707 or 823 (1 × 10^6^) injected directly into the tail vein of 7- to 9-week-old male NOD/SCID mice. Tumor burden was quantified by manually counting nodules visible on the intestinal epithelia surface. 8 week-old NOD/SCID female mice were used for ETHE1 CRC variant xenograft studies.

### Fixation & embedding for electron microscopy

Cells were washed with serum-free media or appropriate buffer, fixed with modified Karmovsky's; 2.5% glutaraldehyde, 4% parafomaldehye and 0.02% picric acid in 0.1M sodium caocdylate buffer at pH 7.2. Secondary fixation in 1% osmium tetroxide, 1.5% potassium ferricyanide samples were dehydrated through a graded ethanol series, and embedded in an epon analog resin. Ultrathin sections were cut using a Leica UltractuS ultramicrotome (Leica, Vienna, Austria) Sections collected on copper grids and contrasted with lead citrate and viewed on a JEM 1400 electron microscope (JEOL, USA, Inc., Peabody, MA) operated at 120 kV. Images were recorded with a Veleta 2K x2K digital camera (Olympus-SIS, Germany).

### Activity of ETHE1 in CRC lysates

ETHE1 consumption of gluathione persulfide (GSSH) experiments were prepared according to reference (20).

### Q-TOF LC-MS and LC-MS/MS

Mass Spectroscopy (MS) was performed as previously performed in [59]

### Proliferation assay

Cells were seeded in 96 well clear plates at 50,000 cells/well. After 24 hrs fresh cell culture media containing 1x Brdu solution (cell biosciences) was added to media. Cell number was determined by measuring absorption at 450nM.

### GSSG and cAMP Assay

Cells were seeded in 96 well white plates 50,000 cells/well. After 24 hrs, cells are lysed to release cAMP or total GSSG (Promega). cAMP or GSSH was determined by Luminesce using a FLUOstar Omega Multi-Mode Microplate Reader with CCD-based Spectrometer.

### Proteomic and ingenuity pathway reanalysis

Reanalysis of the 214 protein spots that were identified as changed in intensity in FAP compared with the control group with p<0.01 and fold changes each > half fold. The David Bioinformatics Gene Ontology at NIH is one of the best accepted software for this analysis. The UNIPROT_ACCESSION names of the up or down proteins in spreadsheet “up vs down in 214” were submitted as gene list to David Bioinformatics site (http://david.abcc.ncifcrf.gov/summary.jsp) for “functional annotation clustering” analysis, using the human genome as background. The software calculates how much of the data fits into each protein category, according to Gene Ontology (GO terms) or KEGG protein pathway database.

## SUPPLEMENTARY MATERIALS FIGURE AND TABLE





## Abbreviations

ETHE1: ethylmalonic encephalopathy 1;
ETHE1-CE: ETHE1 constitutive expression;
CRC: colorectal cancer;
CCIC: colon-cancer-initiating cells;
NOD/SCID: non-obese diabetic/severe combined immunodeficient;
EM: electron micrographs;
