# Supplementary material for: ETHE1 overexpression promotes SIRT1 and PGC1α mediated aerobic glycolysis, oxidative phosphorylation, mitochondrial biogenesis and colorectal cancer
Source: Oncotarget. 2019 Jun 18;10(40):4004–17. doi: 10.18632/oncotarget.26958 (PMC6592291; doi:10.18632/oncotarget.26958)
Supplement: Supplementary file 1 [file oncotarget-10-4004-s001.pdf]

ETHE1 overexpression promotes SIRT1 and PGC1α mediated aerobic glycolysis, oxidative phosphorylation, mitochondrial biogenesis and colorectal cancer

SUPPLEMENTARY MATERIALS

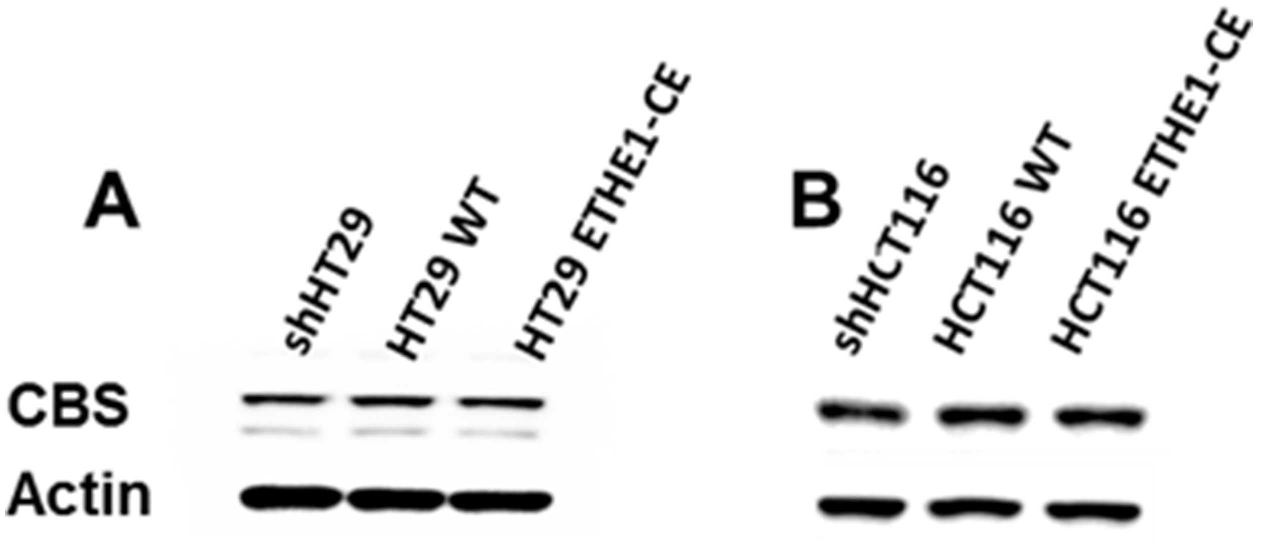

Supplementary Figure 1: CBS expression in **(A)** HT29 and **(B)** HCT116 shRNA knockdown, scrambled control(WT), ETHE1 constitutive expressing cells; Anti-CBS (1:10000 Abcam).

Supplementary Table 1: FAP mitochondrial enrichment correlates with ETHE1 protein levels reanalysis of 214 differential expressed protein spots, comparing FAP to control group, show mitochondrion category is the most prominent up regulated component. 66 Non-redundant proteins (each with p<0.01) were up-regulated more than 0.5 fold in FAP compared to control group. Mitochondria up regulation was prominent in FAP tissues, 30 proteins (row 8 in red, protein id are given), p=1.62E-15 for the identification of this category, and a FDR of the category of only 2.04E-12.

See Supplementary File 1
